# Risk factors associated with the outcomes of fluoroscopy guided pneumatic enema reductions of intussusceptions

**DOI:** 10.4102/sajr.v29i1.3155

**Published:** 2025-09-18

**Authors:** Cornelia M. Kamffer, Hilge du Preez, Jacques Janse van Rensburg

**Affiliations:** 1 Department of Clinical Imaging Sciences, Faculty of Health Sciences, University of the Free State and Universitas Academic Hospital, Bloemfontein, South Africa; 2 Department of Paediatric Surgery, Faculty of Health Sciences, University of the Free State and Universitas Academic Hospital, Bloemfontein, South Africa

**Keywords:** intussusception, paediatric radiology, enema reduction, risk factors, paediatric emergencies, middle income country

## Abstract

**Background:**

Intussusception is a common cause of paediatric bowel obstruction requiring urgent management to prevent ischaemia. Fluoroscopy-guided air enema reduction (FGAR) is the standard non-surgical treatment for uncomplicated cases.

**Objectives:**

To identify factors associated with FGAR outcomes in paediatric intussusception.

**Method:**

A retrospective analytical study was conducted at Universitas Academic Hospital, Bloemfontein, South Africa, including 110 patients with radiologically confirmed intussusception from November 2016 to December 2022. Data encompassed demographics, clinical presentation, laboratory results, and imaging findings.

**Results:**

Among 110 cases (median age 7 months, symptom duration 2 days), 37 were primarily surgically managed, while 73 underwent FGAR attempts (31 successful, 42 unsuccessful, requiring surgery). Of the 79 surgical cases, 24 had manual reduction without resection. Unsuccessful FGAR was significantly associated with younger age (*p* = 0.0249), dehydration (*p* = 0.0299), ascites (*p* = 0.0172), and increased outer wall intussusception diameter on ultrasound (*p* = 0.0026).

**Conclusion:**

In this South African cohort, unsuccessful FGAR was linked to younger age, dehydration, ascites, and larger intussusception size on ultrasound. Early recognition and routine ultrasound use are critical in resource-limited settings to enhance non-surgical outcomes and reduce surgical burden.

**Contribution:**

This study identifies predictors of FGAR failure in a low-resource context, informing clinical decision-making and addressing a gap in the literature on intussusception management in low- and middle-income countries.

## Introduction

Intussusception is a common paediatric abdominal emergency and has been reported to occur in 1–4 out of 2000 children.^1,2^ It is the most common cause of small bowel obstruction in children under the age of 2 years.^1,2^ It requires rapid diagnosis and management to prevent significant morbidity and mortality. An intussusception occurs ‘when a segment of bowel (the intussusceptum) invaginates into an adjacent segment (the intussuscipiens)’.^1,3^ This leads to venous congestion and oedema of the bowel wall. Well-described causes, pathophysiology and positions in which intussusceptions occur exist in the literature.^4^

The internationally recommended standard of management of an uncomplicated intussusception is a non-surgical enema reduction using radiological imaging guidance in the absence of contraindications, with international reported success rates of between 60% and 90%.^5,6^ The absolute contraindications for fluoroscopy-guided air enema reduction (FGAR) are sepsis, peritonitis, shock or haemodynamic instability and bowel perforation.^5^ The total intussusception mortality rates in high-income countries are less than 1% compared to 8.4% – 28% in some low-income countries.^7^

There is still a discrepancy in management between outcomes in high-income countries compared to low-income countries, primarily because of the delay in presentation, diagnosis, and transport to a definitive care centre. A systematic review of 16 intussusception studies in Africa has found that 87% of the reviewed cases were managed surgically, in contrast to what is obtained in high-income countries, where the non-operative successful management rate ranges from 60% to 80%.^8^ Surgical management poses its own unique risk, generally increasing the length of hospital stay and placing a strain on already limited hospital resources.

Although South Africa is classified as a high-middle-income country, the healthcare system’s dichotomy creates challenges similar to those in low-income countries, including delayed diagnoses and transport delays. These factors contribute to delays in initiating treatment, such as rehydration, thereby prolonging the duration of the intussusception, leading to increased vascular compromise of the bowel. Even though resources to manage patients presenting with an intussusception non-operatively at the institution where this study was conducted are available, regular failed enema reductions still occur.

Limited data are available regarding the factors associated with the outcomes of pneumatic reductions of intussusceptions in the South African population. This study aimed to address this gap in the literature. The primary objective was to determine the factors influencing outcomes of fluoroscopy-guided pneumatic reductions of intussusceptions in the paediatric population and to assess the outcomes of patients admitted to this institution with intussusceptions.

## Research methods and design

### Study design and sample

A retrospective analytical study was conducted at a tertiary hospital in Bloemfontein in the Free State province of South Africa. Universitas Academic Hospital is the referral hospital for paediatric surgery services required from referring health care institutes in the Free State province, the Northern Cape province and Lesotho. Patients under the age of 13 years with a radiologically confirmed intussusception from 01 November 2016 to 31 December 2022, were included. Intussusception cases that were already diagnosed and managed at other hospitals and cases with ultrasound features not suggestive of intussusception were excluded.

### Data collection

The Department of Paediatric Surgery’s weekly statistics and ward records were assessed to identify cases and obtain information on admitted cases that met the inclusion criteria. Data were collected from the Paediatric Surgery departmental database, clinical notes on the hospital electronic databases, and diagnostic radiological reports and images stored on the picture archiving and communication system (PACS).

No identifiable data were included, and records were deidentified by allocating a record ID instead of a hospital folder number or patient ID. A REDCap data sheet was used to capture data on clinical history, demographics, clinical presentation, radiological and laboratory findings, management and outcomes of cases. The radiographic findings were interpreted by either a radiology registrar or a paediatric surgery registrar at the time of presentation for each case. Ultrasound examinations were performed and reported by radiology registrars, ranging from the first to the final years of their postgraduate radiology training.

**FIGURE 1:**
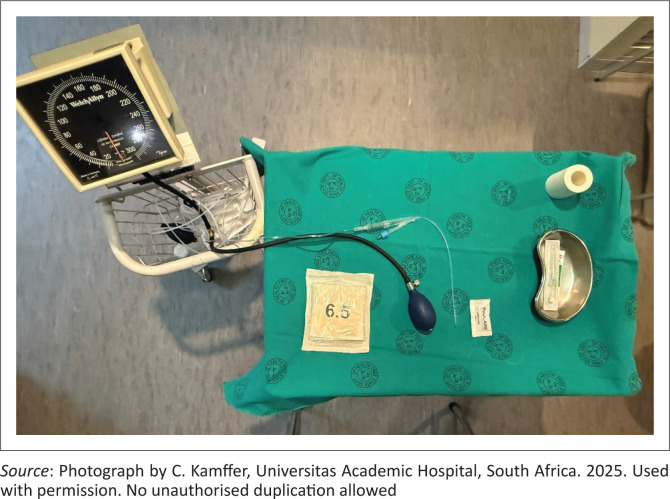
Equipment setup used during fluoroscopy-guided enema reduction at Universitas Academic Hospital.

At Universitas Academic Hospital, non-operative management is carried out using fluoroscopy-guided pneumatic enema reductions. These procedures are performed by radiology registrars, with a paediatric surgeon in attendance, using an equipment setup comprising a Foley’s catheter, lubricating jelly, strapping for the buttocks to secure the Foley’s catheter, an inflation device with a manometer, two large-bore (18 G) intravenous catheters for decompression in case of a tension pneumoperitoneum and gloves, as illustrated in Figure 1.

Pre-procedural sedation is usually not required; however, if analgesia is necessary, it is provided. Definite contraindications to non-operative management include the presence of free extraintestinal air on radiographs, suggesting bowel perforation, as well as sepsis, peritonitis and haemodynamically unstable patients.^6^ In the absence of contraindications, three attempts are conducted at pressures of 80 mmHg, 100 mmHg and 120 mmHg, with a 3-min rest period between attempts. Attempts are repeated three times at each pressure. Our institutional guidelines for an intussusception that does not initially reduce entirely are to re-attempt the reduction after a 3- to 6-h interval period to allow some of the oedema to subside and increase the likelihood of a successful pneumatic reduction. If the pneumatic reduction remains unsuccessful or the patient develops contraindications to a pneumatic reduction, the patient will proceed to surgery. In the absence of contraindication, a third attempt will be made after another 3- to 6-h interval period. These guidelines were used to guide the data collection sheet for the outcomes of the cases in this study.

### Data analysis

The University of the Free State Department of Biostatistics, Faculty of Health Sciences, conducted the statistical analysis with Statistical Analyses Software (SAS 9.4). Medians, minimum and maximum values were summarised as numerical variables, while frequencies and percentages were summarised as categorical variables. For unpaired data, differences between groups and categorical variables were evaluated using appropriate statistical tests (Chi-square or Fisher’s exact test). The Wilcoxon two-sample test for unpaired data evaluated differences between groups and numerical variables.

### Ethical considerations

The University of the Free State Health Sciences Research Ethics Committee provided written approval (reference number: UFS-HSD2022/2056/2305).

## Results

The median age at presentation was 7 months (interquartile range [IQR] 2–48 months). A history of viral-like symptoms within the preceding 2 weeks was reported in 32 of the 110 (29.1%) patients. The most common clinical presenting signs were blood and mucoid stool (red currant jelly stools), which occurred in 97 of the 110 (88.18%) cases. Other clinical presentations are summarised in Table 1.

**TABLE 1:** Common clinical presentations.

Clinical presentation	Cases from the total study sample	
	*n*	%
Bloody and mucoid stool	97	88.18
Bilious vomiting history	60	54.55
Tachycardia	51	46.36
Dehydration	43	39.09
Left-sided abdominal mass	29	26.36
Lethargy	27	24.55
Hypotension	17	11.82
Right-sided abdominal mass	10	9.09
Rectal prolapse of intussusception	5	4.55
Fever	5	4.55

The duration of symptoms ranged from less than 24 h to more than 7 days, with a median duration of 2 days (IQR 1–4 days). All the cases presented with ileocolic intussusceptions.

Primary surgical intervention was performed in 37 (33.64%) cases because of contraindications for pneumatic enema reduction and/or clinical decision based on individual case assessment. Contraindications for pneumatic reduction included shock (*n* = 24; 64.86%), suspected bowel ischaemia (*n* = 17; 45.95%) and peritonitis (*n* = 8; 21.62%). A total of 73 (66.36%) of the cohort qualified for non-operative fluoroscopy-guided pneumatic enema reduction.

**FIGURE 2:**
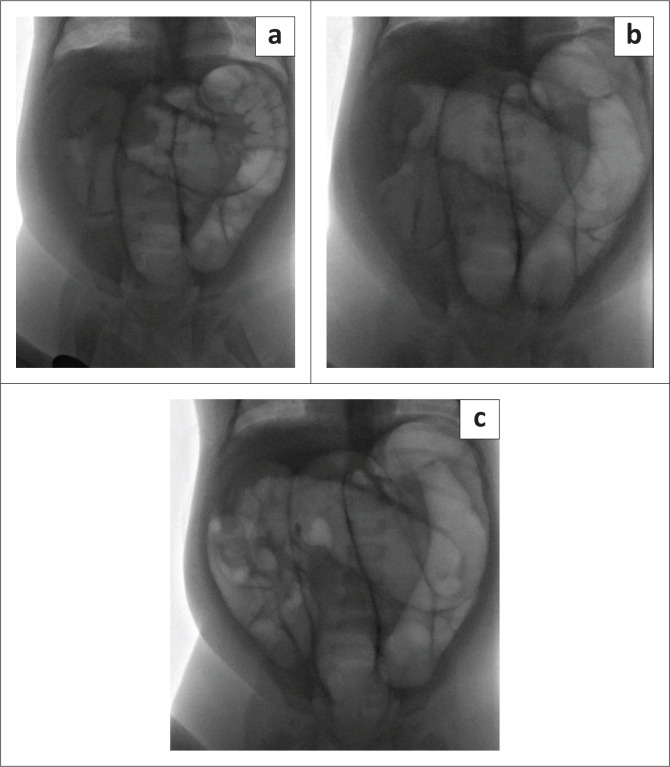
(a–c): A case of an intussusception that was successfully reduced with fluoroscopy-guided air enema reduction (FGAR) after an ultrasound was done, which confirmed an ileocolic intussusception with a ‘target sign’ in the midline upper abdomen. (a) Initially, a soft tissue mass ‘meniscus sign’ can be appreciated within the proximal transverse colon, with minimal air in the small bowel and no signs of perforation. (b) After initial low starting pressure enema at 80 mmHg, one can appreciate slight movement of the ‘meniscus sign’ and/or soft tissue mass at the hepatic flexure. (c) Absence of the ‘meniscus sign’ within the colon or small bowel, with air appreciated within the small bowel after successful reduction at 100 mmHg.

In the cases managed with FGAR, 31 (42.47%) were successful, of which 26 were successful on the first attempt, 5 were reduced after the second attempt, and none were successful on a third attempt. Figure 2 demonstrates fluoroscopic frames from one of the 31 (42.47%) that underwent successful FGAR of an intussusception. There was a median interval time period of 4 h between attempts. Reductions were unsuccessful in 42 of 73 cases (57.53%). Decisions to discontinue further enema reduction and proceed to surgery were made on the basis of three failed attempts of pneumatic enema reduction, without further movement of the intussusception, suspected bowel necrosis and bowel perforation during the attempted pneumatic enema reduction. Repeated attempts, up to a total of 3, were performed in 30 (71%) of the 42 cases.

Bowel perforation occurred in 3 of the 73 (4.1%) cases during the attempted pneumatic enema reduction. Intraoperatively, 48 of the 79 patients (60.76%) who underwent surgery (either primarily or after attempted FGAR) had bowel necrosis, which was managed by resecting the ischaemic bowel and performing a primary anastomosis. A further 6 of the 79 patients (7.59%) had bowel ischaemia intra-operatively, requiring resection of the ischaemic bowel and a temporary stoma. In the remaining 24 of the 79 patients (30.38%), a manual open reduction was performed intraoperatively, and no ischaemic bowel was visualised. In the group where an open surgical reduction was performed, 14 of the patients had undergone an attempted pneumatic reduction, and the remainder presented either with contraindications to pneumatic enema reduction or were managed operatively based on individual assessment and suspected bowel necrosis. In one case, it was documented that the intussusception had self-reduced during the laparotomy.

In the group that had failed the FGAR of the intussusception (*n* = 42), a total of 28 (66.67%) underwent bowel resection because of necrotic bowel found during surgery. In the remaining 14 patients, manual reduction of the intussusception during laparotomy was possible, with no signs of bowel ischaemia. On further analysis, pneumatic reduction was attempted only once in seven cases, twice in three cases and thrice in five cases. Thereafter, the patients proceeded to surgical management with no specific contraindication present. In three of the patients who were part of the unsuccessful group, a pathological lead point was found, including Meckel’s diverticulum and Wauch syndrome.

In both the successful and unsuccessful pneumatic reduction groups, there was a median duration of symptoms of 2 days before presentation. However, when comparing the successful FGAR group with the primary surgical-managed group, there was a statistically significant (*p*-value 0.0007) difference in the duration of symptoms between these groups, where the median duration of symptoms in the primary surgically managed group was 3 days with an upper quartile of 4 days compared to 2 days in the successful FGAR group. Table 2 provides a detailed summary of the clinical and demographic presentation of the successful FGAR compared to the unsuccessful group; of these parameters, only age at presentation and dehydration were statistically significant.

**TABLE 2:** Demographic and clinical presentation of the successful fluoroscopy-guided pneumatic reduction group compared to the unsuccessful group.

Variable	Successful group (*n* = 31)		Unsuccessful group (*n* = 42)		*p*
	*n*	%	*n*	%	
Median age at presentation (months)	7	-	5.5	-	0.0249*
Preceding viral history	5	16.00	15	35.71	0.0576
Bilious vomiting history	13	41.94	20	47.62	0.6296
Bloody and mucoid stool (red currant jelly stool)	29	93.55	37	88.10	0.6912
Tachycardia	7	22.58	17	40.48	0.1077
Dehydration	3	9.68	13	30.95	0.0299*
Left-sided abdominal mass	6	19.35	12	28.57	0.3665
Right-sided abdominal mass	2	6.45	2	4.76	1.0000
Lethargy	3	9.68	9	21.43	0.1806
Hypotension	1	3.23	3	7.14	0.6322
Fever	3	9.68	0	0.00	0.0723
Rectal prolapse of intussusception	1	3.23	3	7.14	0.6322
Non-bilious vomiting	9	29.03	10	23.81	0.6152
Median duration of symptoms (days)	2	-	2	-	0.0628

* , *p* value of < 0.05 is significant.

The abdominal radiograph findings most commonly described in the literature for cases of intussusception were included in this study. However, no significant statistical associations were found between these findings and the outcome of fluoroscopy-guided pneumatic enema reduction in this population. As seen in Table 3, there was no statistically significant difference in plain X-ray findings between the groups in this study.

**TABLE 3:** Plain film findings of the successful fluoroscopy-guided pneumatic reduction of the intussusception group and the unsuccessful group.

Variable	Successful group (*n* = 19)		Unsuccessful group (*n* = 30)		*p*
	*n*	%	*n*	%	
Free air under the diaphragm	0	0.00	0	0.00	-
Meniscus or target sign	0	0.00	0	0.00	-
Signs of bowel obstruction	11	57.89	19	63.33	0.7034*
Pneumatosis intestinalis	0	0.00	0	0.00	-
Dilated bowel loops (non-specific)	5	26.32	7	23.33	1.0000*
Normal	3	15.79	4	13.33	1.0000*

* , *p*-value of < 0.05 is significant.

The presence of ascites and an outer wall-to-outer wall diameter of more than 30 mm on ultrasound were associated with an unsuccessful FGAR outcome, as summarised in Table 4. The authors also evaluated the C-reactive protein (CRP) and white cell counts (WCC) as markers of sepsis. However, they found no predictive value in their elevated levels regarding the success of pneumatic reduction (CRP *p*-value 0.6152 and WCC *p*-value 0.1655).

**TABLE 4:** Comparison of ultrasound findings between the successful fluoroscopy-guided air enema reduction (FGAR) group and the unsuccessful FGAR group.

Variable	Successful group (*n* = 31)		Unsuccessful group (*n* = 40)		*p*
	*n*	%	*n*	%	
Free fluid in the abdomen/ascites	5	16.13	17	42.50	0.0172*
Intussusception was noted on the right side of the abdomen	12	38.71	13	32.50	0.6634
Intussusception was noted on the left side of the abdomen	13	41.93	19	47.50	0.5355
Intussusception was noted in the midline of the abdomen	5	-	7	-	0.8538
Maximum outer wall-to-outer wall diameter (transverse view) median in mm	26	-	30	-	0.0026*

* , *p*-value of < 0.05 is significant.

A total of six cases of recurrence occurred in the successful pneumatic enema reduction group; of these, five cases reoccurred within 48 h post-pneumatic enema. In each of these five cases, a repeat pneumatic enema reduction was successful on the first attempt, and no subsequent recurrences were documented. The longest interval between recurrences was in an 18-month-old patient who experienced a recurrent intussusception 2 months after the initial presentation. This was successfully reduced on the first attempt, but another recurrence occurred 48 h later. The patient was then taken to the theatre for a suspected pathological lead point, which was subsequently found (Waugh syndrome with atypical malrotation), and a Ladd’s procedure had to be performed.

The two mortalities in this study (1.82% mortality rate) included a case where extensive mesenteric thrombosis was present and another in which the initial clinical presentation was hypovolemic shock and sepsis with multiple cardiac arrests before demise.

Post-operative complications documented in the study for the unsuccessful FGAR group included three patients with hospital-acquired infections, one patient with post-surgical abdominal collections, and three patients who required re-look laparotomies for suspected bowel necrosis (one) and anastomotic leaks (two). There was a statistically significant correlation in the length of stay between the successful fluoroscopy-guided pneumatic enema group and the unsuccessful group (*p* < 0.0001), with a median hospital stay duration of 2 days in the successful group compared to 6 days in the unsuccessful group.

## Discussion

The median age at presentation in this study was 7 months, which is in keeping with the literature describing 5 months of age as the usual age of presentation, with a rise in incidence between 4 and 9 months and a lower incidence after 18 months.^4^ Furthermore, the median age of presentation influenced the outcome of fluoroscopy-guided pneumatic enema, as younger patients (with a median age of five and a half months old) were more likely to fall into the unsuccessful group (*p*-value 0.024). The literature suggests an age of less than 1 year at presentation as a risk factor for a failed enema reduction.^6^ Clinical and demographic risk factors associated with failed enema reductions are prolonged symptoms, intussusception in the distal colon, diarrhoea, vomiting, rectal bleeding, younger patients and signs of small bowel obstruction.^6^ Although no specific value is known, it is well established that a longer duration of symptoms is associated with an increased risk of failed enema reduction.^6^ This study could not demonstrate a statistically significant difference between the successful and unsuccessful groups in the duration of symptoms; however, a significant difference was observed in the duration of symptoms between the primary surgically managed and successful FGAR groups (*p*-value 0.0007), where the median duration of symptoms in the primary surgically managed group was 3 days, with an upper quartile of 4 days, compared to 2 days in the successful FGAR group.

In this study, a preceding viral history was more common in the unsuccessful group (35.71%) compared to 16% in the successful group. This could be owing to underreporting of history and is considered a study limitation because of the retrospective data collection. A relation between some enteric pathogens and intussusceptions has been described, specifically gastroenteritis and upper respiratory tract infections.^9^ The only statistically significant difference in clinical presenting symptoms and signs between the successful and unsuccessful groups in this study was dehydration. In both groups, the most common clinical presentations were bloody mucoid stools and bilious vomiting. The only cases with reported fever were in the successful group, suggesting that fever is not necessarily a predictor of unsuccessful reduction, as supported by the literature.

On supine and erect plain abdominal films, two specific signs of intussusception can be observed, namely the ‘target’ and ‘meniscus sign’.^10^ The ‘target sign’ is observed because of the mesenteric fat dragged into the intussusception and can be appreciated as a round soft tissue mass with a concentric lucency.^10^ The ‘meniscus sign’ is the observation of a crescent gas in the colonic lumen that outlines the intussusception apex.^10^ Although these findings are described in the literature, they were not routinely documented in this study or readily observed, and abdominal radiographs should be reserved for cases of suspected perforation because of the low sensitivity.^11^

**FIGURE 3:**
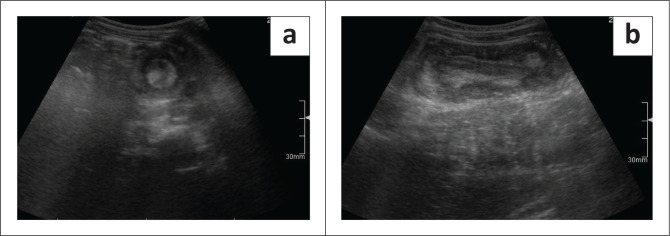
(a) The doughnut or bull’s eye or target sign is a pathognomonic transverse-view ultrasound finding and, in the clinical suspicion of intussusception, is highly sensitive and specific. (b) The pseudo kidney sign describes the appearance on the longitudinal ultrasound view of an intussusception.

An abdominal ultrasound is the primary imaging modality for diagnosing an intussusception and has a 100% negative predictive value and high sensitivity and specificity values of 98% – 100% and 88%, respectively.^11^ On transverse imaging, a soft tissue mass – the ‘doughnut sign’, ‘target sign’, ‘multiple concentric ring sign’ and ‘crescent in doughnut sign’ – appears as a central hyperechoic core (the intussusceptum) and a hypoechoic outer rim of homogeneous tissue (the intussuscipiens), as demonstrated in Figure 3a.^11^ Longitudinal findings are the ‘pseudo kidney sign’, ‘the sandwich sign’ and ‘hayfork sign’, synonymous terms used to describe the observation of a hyperechoic tubular centre covered by a hypoechoic rim, producing the signature kidney resemblance, as demonstrated in Figure 3b.^11^

One of the possible predictive signs of an irreducible intussusception on ultrasound that has been described is the absence of colour Doppler flow on ultrasound, which could suggest bowel ischaemia.^11^ Evaluation of this sign was excluded from this study because of operator-dependent images and inconsistent inclusions in the imaging report.

Two significant ultrasound findings were the presence of ascites and the outer wall diameter on ultrasound, where the unsuccessful FGAR group had a larger median outer wall transverse ultrasound diameter of 30 mm compared to 26 mm in the successful group. Outer wall diameter in intussusception has been studied by Kim et al., who found that there is a larger outer wall diameter in complicated small bowel intussusceptions (mean outer diameter was 2.9 cm [range: from 2 cm to 4.3 cm]) and in the ileocolic intussusceptions (3–5 cm), which more frequently required surgery compared to transient small bowel intussusceptions (< 2.5 cm). However, at the time of this report, the authors could not find any other literature available on the association between outer wall diameter of an intussusception on ultrasound and the outcome of FGAR specifically for ileocolic intussusceptions, as in this study.^12^ When examining further investigations to correlate outer wall diameter with duration of symptoms with regards to outcomes, this study could not identify a Spearman Correlation Coefficient that was statistically significant when comparing the duration of symptoms with the outer wall-to-outer wall ultrasound diameter of an intussusception. A more robust study is needed, incorporating a larger sample size and enhanced control over operator variability to ensure greater reliability and validity of this finding, address the surrounding limitations and possibly use this as a predictor of FGAR outcome.

There was a statistically significant difference between the two groups regarding ascites detected on ultrasound. This is in keeping with the literature that ascites has been reported as an ultrasound finding associated with failed pneumatic reduction because of prolonged increased venous pressure and venous congestion.^5^ This study found no correlation between the side of the intussusception and the outcome of FGAR.

This study did not find a statistically significant difference in the WCC and CRP values between the successful and unsuccessful pneumatic enema reduction groups. However, the median values were higher in the unsuccessful group. Although this was not statistically proven, an assumption can be made that because of raised infective markers indicating either bowel obstruction or ischemic bowel, patients with significantly elevated septic markers will more likely fall into the primarily surgically managed group with contraindications to pneumatic enema reduction.

**FIGURE 4:**
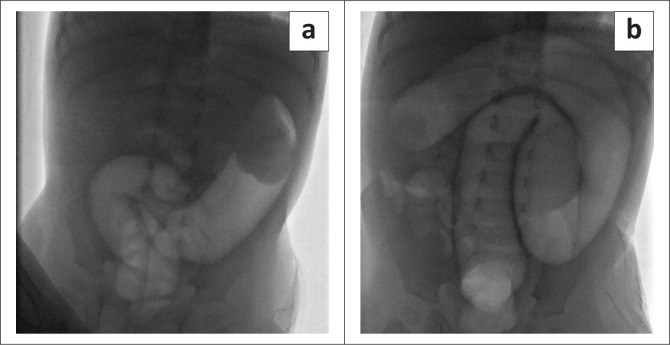
(a, b) A surgically managed case after three failed attempts of pneumatic reduction because of further failure of movement of an ileocolic intussusception. Initially, the intussusception was appreciated near the splenic flexure and moved during the reduction to the hepatic flexure, after which no further movement was appreciated. Ischaemic bowel was found during surgery.

Ten of the 42 cases required manual surgical reduction after one attempt of FGAR and demonstrated no bowel necrosis. The decision not to pursue further reduction attempts was based on a consultant assessment of individual cases with a high clinical suspicion of bowel ischaemia. This raised the question of whether a successful enema reduction would have been achieved if more attempts at pneumatic enema reduction had been made, considering the number of cases that were manually reduced intraoperatively without ischaemia and the need for bowel resection. Surgical management could have possibly been avoided if more attempts were made, as there is no absolute limit on the number of attempted reductions in the presence of haemodynamic stability and the absence of perforation and peritonitis.^13^ Studies have shown that in many cases of a failed first attempt at non-surgical enema reduction, the intussusception reduced spontaneously during surgery or easily reduced manually during surgery.^9^ This advocates for more aggressive non-surgical management approaches and, therefore, reserves surgery only for cases with obvious necrotic bowel or contraindications to enema reduction (Figure 4).^14^

Cases of recurrent intussusceptions are more often associated with pathological lead points.^4^ This study recorded six (5.5%) cases of recurrent intussusception similar to the 8% – 15% described in the literature.^13^ No definite guidelines exist for managing recurrent intussusception, but the literature suggests managing these cases as an index presentation.^11^ Surgical reduction should be considered in cases of failed enema reduction when a pathological lead point is suspected or in cases of several recurrent episodes.^11^ The exact number of recurrences allowed before operative intervention is not clearly described. However, some studies have suggested that operative intervention is indicated after three recurrences.^13^

In this study, three documented cases of bowel perforation during attempted pneumatic enema reduction led to a perforation rate of 4.1%, in keeping with the 0% – 5% described in the literature.^15^ Two cases perforated during the first attempt of FGAR at a pressure of 120 mmHg and one during the second attempt at 80 mmHg. None of these cases presented clinically ill, had contraindications for FGAR or had a specific clinical history to suggest possible complications during enema reduction. Thus, perforation was an unexpected and unpredictable complication necessitating management as per our protocol.

The shorter hospital stay in the successful FGAR group was expected, as surgically managed patients tend to have a prolonged hospital stay compared to non-surgically managed patients. Increased hospitalisation poses unique challenges related to increased risk of hospital-acquired infections, increased healthcare cost, increased burden on a healthcare system, and the psychosocial impact of hospitalised children on both caregivers and children. A paradigm shift is occurring regarding the duration of stay of uncomplicated intussusception, with some studies suggesting same-day discharge and outpatient management of these cases.^9^ Several other studies found no difference between the recurrence rate and adverse outcomes between patients discharged from an Emergency Department and those managed as inpatients.^14^ Factors that need to be explicitly considered locally are patients’ socioeconomic resources and their ability to readily return to the hospital if complications or recurrence occur.

This study cohort revealed a mortality rate of 1.82%, comparable to that in high-income countries, where the rate is typically less than 1%.^15^ In contrast, low-income countries report higher mortality rates, ranging from 8.4% to 28%.^7^ Despite international standards and guidelines, there is still a large discrepancy in morbidity and mortality rates between low- and high-income countries.^7^ This can mainly be attributed to delays in presentation, diagnosis, transport to definitive care institutions and the lack of medical expertise outside tertiary care centres.^7^ These delays and lack of expertise can cause a delay in definitive management and have been associated with a higher need for primary surgical management, therefore resulting in a higher mortality rate.^7^

### Limitations

This study was conducted at a single centre and was retrospective in nature. The data collected depended on the availability, completeness and accuracy of the clinical notes. Radiological findings were interpreted by Radiology and Paediatric Surgery registrars and not by consultants. Ultrasounds are operator-dependent and influenced by the skill and expertise of the individual performing the scan. Although the department has a standardised protocol for pneumatic intussusception reduction, the variability in the proficiency and experience of the person performing the pneumatic reduction likely also influenced the results.

## Conclusion

This study provides insights into the factors associated with unsuccessful FGAR of intussusception in the South African paediatric population. Younger age at presentation, dehydration, ascites and an increased outer wall-to-outer wall intussusception diameter on ultrasound were significant factors associated with unsuccessful FGAR of intussusception and can be used for clinical decision-making. The fact that several intussusceptions following failed FGAR could be manually reduced intraoperatively advocates for more aggressive attempted FGAR in the absence of contraindications. Reducing the need for surgical intervention reduces the risk of intra- and postoperative complications and should generally reduce the length of hospitalisation and improve patient outcomes.

This study contributes to the limited body of literature on intussusception management in low- and middle-income countries, addressing a critical gap in content-specific paediatric surgical care. These findings underscore the need of early recognition and referral of intussusception cases, alongside the integration of ultrasound as a routine diagnostic tool in resource-limited settings. Implementing these insights into clinical practice can enhance non-surgical treatment outcomes, reduce the burden on surgical services and improve paediatric care in South Africa.
